# Giant left atrium associated with massive thrombus formation

**DOI:** 10.1186/1477-9560-11-5

**Published:** 2013-03-04

**Authors:** Ahmad K Darwazah, Hamdy El Sayed

**Affiliations:** 1 Heliopolis Cardiac Center, 46 Nazeeh Khalefa St. Heliopolis, Cairo, Egypt

**Keywords:** Giant left atrium, Left atrial thrombus, Thrombectomy, Mitral valve replacement

## Abstract

Giant left atrium is a condition characterized by huge enlargement of the left atrium with a diameter exceeding 65mm. It is most commonly associated with long standing rheumatic mitral valve disease. We present a 45-year-old female patient with rheumatic mitral stenosis associated with giant left atrium occupied by an 11 × 10 × 5 cm thrombus weighing 500 gms. The patient underwent successful mitral valve replacement and thrombectomy through an inverted T-shaped biatrial incision.

## Background

Giant left atrium (GLA) is commonly associated with long standing rheumatic mitral valve regurgitation or mixed mitral disease with predominant regurgitation [1]. The exact etiology is not known. Both increased left atrial pressure and weakening of left atrial wall by rheumatic pancarditis are implicated in its development [1,2].

The condition can be associated with atrial fibrillation, thromboembolic complications, hemodynamic and respiratory complications [3]. We present a case of GLA with predominant mitral stenosis associated with atrial fibrillation and huge thrombus formation. The patient was successfully managed by mitral valve replacement and removal of LA thrombus.

## Case presentation

A 45-year-old woman with a known history of rheumatic mitral stenosis presented with a 9 months history of progressive shortness of breath and bilateral peripheral oedema.

The patient had a successful closed mitral valvotomy in 1987, from which she completely recovered and was maintained on digoxin and warfarin.

Few months before admission her activity became limited due to increase shortness of breath, repeated coughing and recurrent paroxysmal nocturnal dyspnea. She was treated with aggressive diuretic regimen, digoxin, cordarone, beta-blockers and warfarin.

Physical examination revealed signs of right sided heart failure with decrease air entry along both lung bases. A mid-diastolic murmur was heard at the apex together with pansystolic murmur at left lower parasternal region.

**Figure 1:**
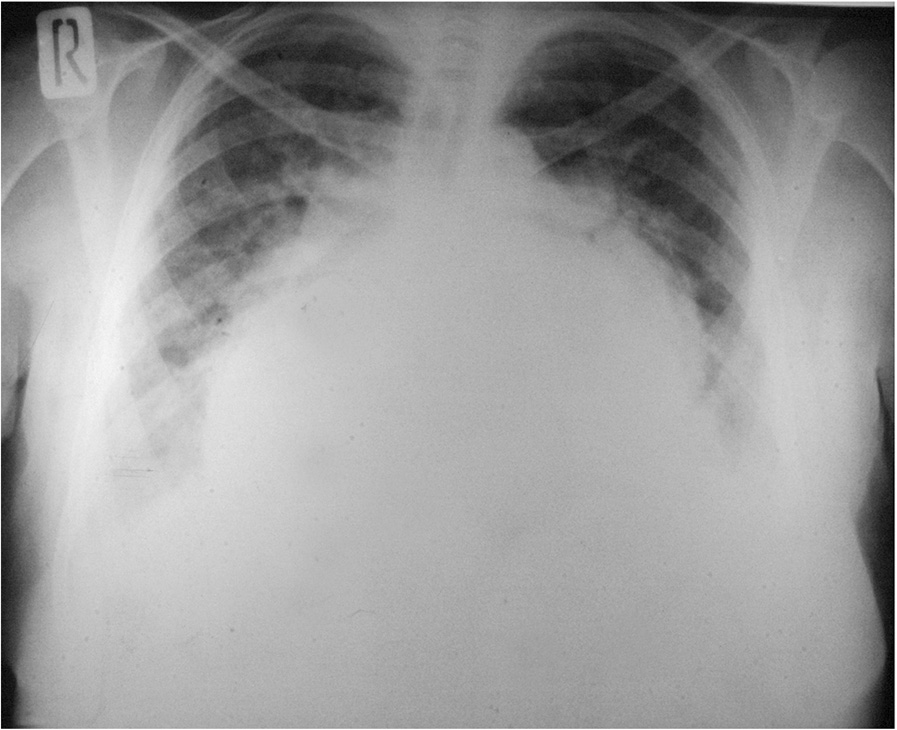
Preoperative chest x-ray showing marked cardiomegally and widening of the carina.

**Figure 2:**
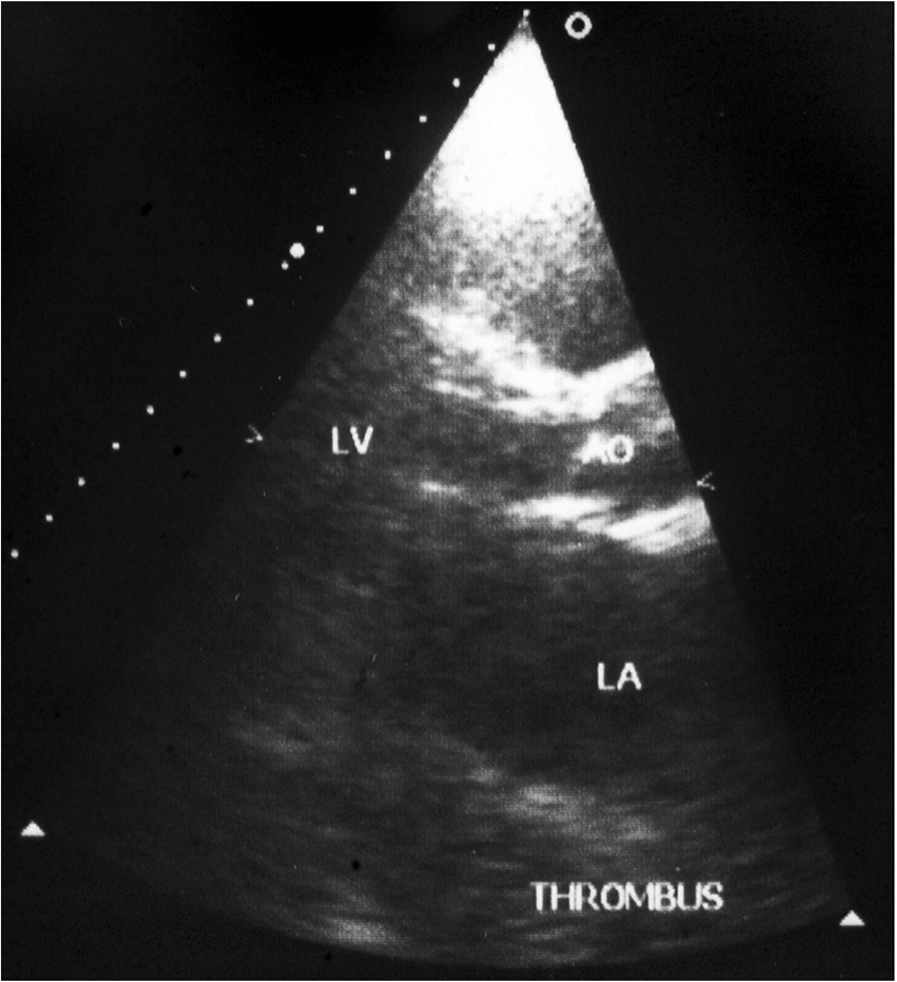
Preoperative echocardiography showing a huge left atrium occupied by a thrombus.

Electrocardiography showed atrial fibrillation with enlarged right ventricle. Marked cardiomegaly was seen on chest radiography (Figure 1). A hugely enlarged left atrium (11 × 10 cm) occupied by a thrombus was demonstrated by transthoracic echocardiography (Figure 2). Evidence of mitral restenosis (MVA 0.9 cm^2^) with mild regurgitation was seen. Tricuspid valve was severely incompetent with an estimated PAP of 75 mmHg.

Laboratory investigations revealed a low hemoglobin level (9.5 g/dL), low platelets count (135,000/cmm), mildly elevated liver enzymes with positive hepatitis C antibodies.

Abdominal ultrasound showed hepatomegaly with mild cirrhosis as well as mild splenomegaly.

**Figure 3:**
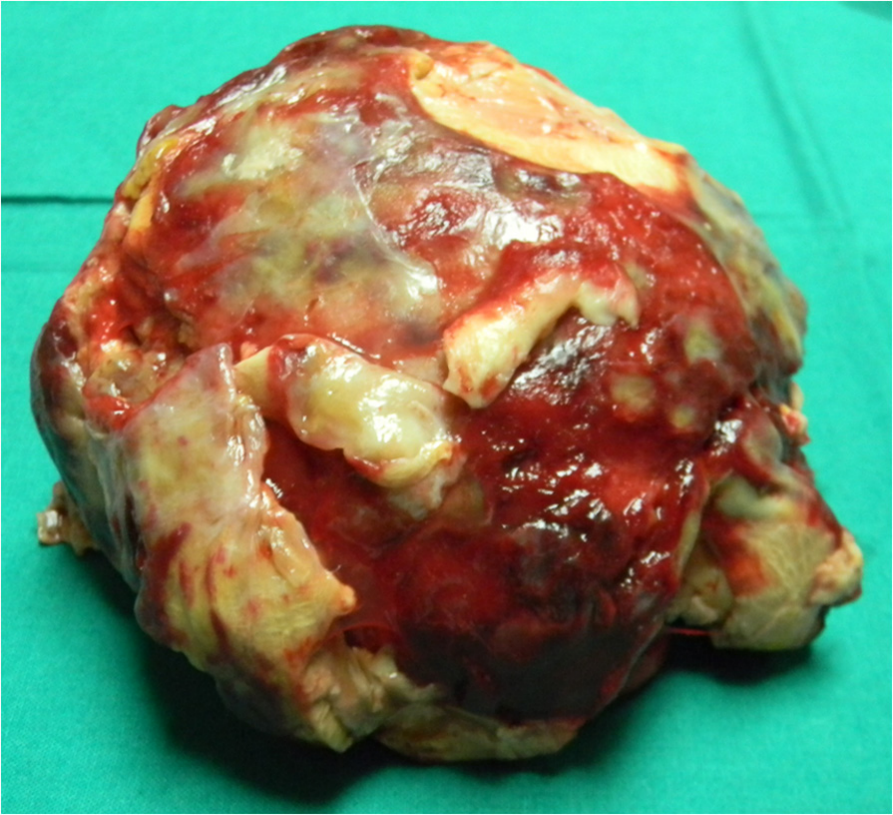
A thrombus measuring 11 x 10 x 5 cm removed from the left atrium.

Surgery was performed using standard CPB with moderate hypothermia and antegrade blood cardioplegia. The stenosed mitral valve was exposed through an inverted T-shaped biatrial incision. A huge organized thrombus measuring 11 × 10 × 5 cm was removed (Figure 3). Mitral valve was replaced with St. Jude Medical valve 31-mm. Tricuspid valve was repaired using Devag’s annuloplasty. Minimal inotropic support was used during weaning from bypass.

Postoperatively, the patient remained hemodynamically stable. She was ventilated for 17 hours and had minimal blood loss. She stayed in ICU for 3 days, during which intensive chest physiotherapy was performed. The patient was discharged after 12 days.

**Figure 4:**
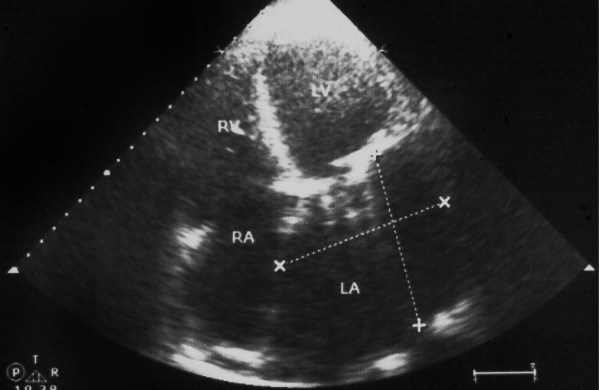
Postoperative echocardiography showing a reduction of left atrial size.

**Figure 5:**
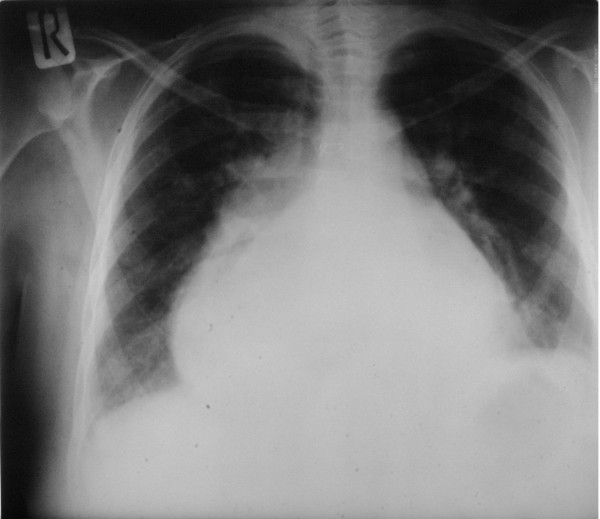
Postoperative chest x-ray showing reduction of cardiothoracic ratio.

Follow-up by TTE and chest X-ray two weeks after surgery showed a well functioning prosthetic valve, clear left atrium with an estimated size of 9 × 9.8 cm (Figure 4). Both lung fields were clear with reduced cardiothoracic ratio (Figure 5).

## Discussion

Long standing mitral valve disease is associated with enlargement of the left atrium as a compensatory mechanism due to increase intracavitary pressure and volume. Such an enlargement is beneficial as it reduces pulmonary congestion, thus it protects the lung from pulmonary hypertension and oedema [4]. However, with gradual increase in left atrial pressure an associated increase in pulmonary venous pressure will eventually occur [2].

Extreme enlargement of the left atrium more than 65mm is known as giant left atrium (GLA) [3]. Its exact etiology is still unknown. The condition has been closely related to rheumatic mitral valve regurgitation or mixed mitral valve disease causing severe pressure and volume overload [1]. However, the condition can be associated with mitral valve prolapse, heart failure, chronic atrial fibrillation and left to right shunt [2,3,5]. Rarely, it can be seen in patients with normal mitral valve function [2].

The mechanism of formation of GLA is not fully understood. Patients with chronic mitral valve disease are not always associated with GLA. Only 19% may develop such a condition [6]. Previous studies showed that chronic pressure in the left atrium is not the only cause of GLA, but weakening of the left atrial wall by rheumatic pancarditis causing chronic inflammation and fibrosis is also implicated [1-3].

Enlargement of the left atrium is associated with development of atrial fibrillation, which in return can lead to further enlargement of left atrium. Such an observation was seen among patients who had atrial fibrillation with absence of cardiac pathology [7].

Huge enlargement of the left atrium is prone to develope various complications including thrombus formation, thromboembolic events, hemodynamic derangements and sudden death [3,8].

Patients with GLA are usually presented with symptoms related to mitral valve disease. Specific symptoms may occur due to compression of the oesophagus and airway by the enlarged posterior wall of the left atrium causing dysphagia and respiratory dysfunction. Cardiac output may be reduced by obstruction of inferior vena cava due to displacement of atrial septum and compression of the postero-lateral wall of the left ventricle by the enlarged left atrium [8]. In rare situations, patients are completely asymptomatic [9].

The enlarged left atrium is associated with blood stasis and thrombus formation. The risk of thrombo embolism increases with left atrial size regardless of anticoagulation [8]. Previous studies found that the incidence of left atrial thrombus among patients with mitral stenosis associated with AF varies from 7-38%. Such an incidence is directly related to the size of left atrium [6,10].

It is interesting to note that not all patients with GLA have an associated thrombus formation [11,12]. Also the size of thrombus varies from one patient to the other [9,13]. Such a diversity can be explained by various local factors in the left atrium.

Goldsmith and colleagues [14], found that patients with mitral valve disease have associated left atrial endocardial damage. They found that severe damage was encountered among patients with mitral stenosis associated with AF. Such damage contributed to the risk of thrombus formation. The other factor is related to the activation of coagulation system within the left atrium [15]. Patients with mitral stenosis have a significantly high level of fibrinopeptide A, thrombin-antithrombin III complex and Von Willebrand factor antigen in the left atrium. These biochemical markers are responsible for the formation of thrombosis even during anticoagulation regardless of the severity of mitral stenosis or size of left atrium [15].

Our patient had a longstanding mitral stenosis associated with chronic AF. Both factors were responsible for the development of GLA and thrombosis. Despite the high incidence of rheumatic valvular heart disease in Egypt, GLA is seen in only 3-4% of our patients. Such a low incidence is probably related to early development of pulmonary hypertension and its subsequent effect on reducing right ventricular output, so both pressure and volume load in left atrium are reduced.

The size of thrombus in our patient was huge in comparison to previously reported cases despite the fact, that the size of the left atrium in our patient was smaller. It is important to mention that the development of thrombosis in our patient occurred despite therapeutic anticoagulation and without any evidence of interruption of oral anticoagulant. These observations agree with previous studies which emphasized the importance of local factors within the left atrium.

Controversies still exist regarding surgical management of GLA by atrial plication. Previous studies showed that plication at the time of mitral valve surgery is essential to reduce left atrial size to eliminate its compressing symptoms and thromboembolic complications^(3)^. However, such a procedure is associated with various complications as circumflex coronary artery injury, pulmonary vein obstruction and oesophageal stricture [16].

A previous study by Tonguc and colleagues [16], compared patients undergoing mitral valve replacement (MVR) with or without plication, found no significant difference in hemodynamic improvement and reduction of left atrial diameter especially when the diameter was below 80 mm.

Also, the incidence of left atrial thrombus after MVR showed no difference whether plication was performed or not [17]. Plication of the left atrium was not performed in our patient, to avoid unnecessary complications. Adhesions from previous surgery, postoperative bleeding due to low platelet count, history of hepatitis C virus and cirrhotic liver were our main concern.

We agree with other investigators [8], that the size of left atrium will not change significantly after MVR due to irreversible damage of left atrial muscle by fibrosis. But we believe that hemodynamics will improve after MVR with or without plication.

The most striking compressive symptom in our patient was on the respiratory system. Although, the size of the left atrium did not change much in the early postoperative period, the reduction in the left atrial pressure and pulmonary artery pressure were sufficient to alleviate the respiratory symptoms. Postoperative chest X-ray showed significant improvement of both lung fields and widening of the carina.

Removal of an organized thrombus from the left atrium can be challenging especially when it is huge in size. The presence of dense adhesions and absence of cleavage plane makes its removal difficult [18]. Under these circumstances, residual organized material can be left. Lim and colleagues [18], suggested the use of autologous pericardial patch to cover these areas to avoid future thrombus formation.

Thrombectomy in our patients was easy to perform despite the huge size. This was facilitated by performing an inverted T-shaped biatrial incision. A line of cleavage was identified and the whole thrombus was completely removed intact.

Early postoperative anticoagulation was of a great concern in the present case. Our patient had multiple factors which can predispose to early thrombosis, among which are, the giant left atrium, associated atrial fibrillation, rough left atrial cavity after thrombectomy and finally the newly implanted mechanical mitral prosthesis.

Various strategies of using early postoperative oral anticoagulation with or without heparin have been reported, but the optimal method to reduce the incidence of thrombosis and to avoid hemorrhagic complications is still debatable.

Oral anticoagulants alone seems to be a safe approach to reduce thrombosis and bleeding [19]. However, such medications are not ideal for early use since several days are usually required to achieve therapeutic INR. The addition of heparin within 24 hours after surgery in combination with oral anticoagulants was recommended to cover the early critical period after valve implantation [20].

Comparing the effect of various types of heparin in relation to thrombosis and bleeding, various studies found that the use of intravenous unfractionated heparin have the highest incidence in comparison to prophylactic dose of sc unfractionated or LMW heparin [21].

Concerning our present case, a single dose of intravenous unfractionated heparin 5000 u was used few hours after surgery after checking coagulation profile and chest drains for bleeding. Oral Coumadin 5 mg loading dose and aspirin 80 mg together with sc prophylactic dose of LMW heparin(clexane) were commenced on the first day posoperatively. Once therapeutic INR (2.5-3) was reached, heparin was discontinued.

Our policy to use intravenous unfractionated early postoperatively instead of LMW heparin, is to be able to control any possible risk of bleeding which might occur. Unfractionated heparin can be fully reversed by protamine when compared with LMW heparin.

The addition of low dose of aspirin with oral anticoagulants potentiates the reduction of thromboembolism [21], thus reducing the dose of oral anticoagulants and the incidence of bleeding complications among these patients.

## Conclusions

GLA can be associated with huge thrombus formation. Various factors are implicated in the formation of thrombosis, among which the local left atrial factors seem to play a major role. Complete removal of huge thrombus can be facilitated by the use of an inverted T- shaped biatrial incision.

### Consent

Written informed consent was obtained from the patient for publication of this case report and any accompanying images. A copy of the written consent is available for review by the Editor-in- Chief of this journal.

## Competing interests

The authors declare that they have no competing interests.

## Authors’ contributions

Both authors were involved in the management of the patient. AKD carried out the operation and writing the manuscript. HE provided his experience in the subject and follow up of the patient. Both authors read and approved the final manuscript.
